# Investigating the Impact of Cold Agglutinins on Red Blood Cell Parameters in a Trauma Patient

**DOI:** 10.7759/cureus.68379

**Published:** 2024-09-01

**Authors:** Tirath Patel, Rohab Sohail, Hanyie Chang, Michelle Addo, Richard M Millis

**Affiliations:** 1 Department of Medical Physiology, American University of Antigua, St. John’s, ATG; 2 Department of Internal Medicine, Bayhealth Medical Center, Dover, USA; 3 Department of Medical Physiology, American University of Antigua, St. John's, ATG

**Keywords:** mean corpuscular volume, mean corpuscular hemoglobin, hematocrit, red blood cell disorder, clinical hematology, cold agglutinins, traumatic injury

## Abstract

Cold agglutinins are autoantibodies that can cause primary hemolytic anemia and RBC agglutination syndrome. Secondary agglutination of RBCs may be found in hypothermia, as well as in cancers, infections, and traumatic injuries. This report presents the case of a 37-year-old man who suffered multiple injuries in a motorcycle accident. On admission, the patient’s laboratory tests showed a high concentration of cold agglutinins associated with low RBC count, hemoglobin, and hematocrit, and elevated mean corpuscular hemoglobin and mean corpuscular volume. Intravenous immunoglobulin treatment was effective at reversing the abnormal blood parameters to normal. Unlike acute blood loss, which typically manifests with normal hemoglobin and hematocrit levels initially due to proportional loss of plasma and red cells, the presence of cold agglutinins can lead to abnormal agglutination and sequestration of RBCs, with low hemoglobin and hematocrit. The findings of this case report highlight the importance of recognizing cold agglutinins in trauma patients to avoid misdiagnosis and misinterpretation of laboratory results.

## Introduction

Cold agglutinins cause agglutination of RBCs at temperatures below the normal body temperature. These autoantibodies are commonly associated with viral infections and autoimmune disorders [1]. In trauma patients, cold agglutinins can be produced in response to the release of inflammatory cytokines [1]. Trauma is known to be a significant cause of morbidity and mortality worldwide, and patients with traumatic injuries often require emergency care. The diagnosis and management of these patients require prompt and accurate laboratory testing to guide appropriate interventions [2]. The impact of cold agglutinins on RBC parameters in trauma patients is not well understood. The presence of cold agglutinins may cause hemolysis, leading to a range of clinical symptoms such as fatigue, weakness, and shortness of breath. These symptoms are also characteristic of anemia associated with acute hemorrhage, a frequent complication of traumatic injury. The impact of cold agglutinins on RBC parameters in trauma patients can, therefore, result in misinterpretation of laboratory results, delay in diagnosis, and inappropriate management [3-5]. The purpose of the present case report is, therefore, to demonstrate the impact of cold agglutinins on RBC parameters in a trauma patient, with an emphasis on the knowledge required to avoid misdiagnosis and ensure prompt, effective treatment.

An abbreviated (draft) version of this case report can be found on the Authorea preprint server (doi: 10.22541/au.168011867.72979893/v2).

## Case presentation

A 37-year-old male presented to the emergency department following a motorcycle accident, appearing somnolent but arousable, oriented, and intoxicated, with a Glasgow Coma Scale (GCS) score of 15 (E4V5M6). He complained of difficulty breathing, speaking in short sentences, and gasping for air. The patient reported drinking several beers but denied drug use, loss of consciousness, or vomiting. Vital signs indicated elevated blood pressure and heart rate, with low respiratory rate and oxygen saturation. Physical examination revealed multiple injuries, including a scalp laceration, fractured ribs, and a fractured leg, with no significant past medical or surgical history.

Laboratory investigations

**Figure 1:**
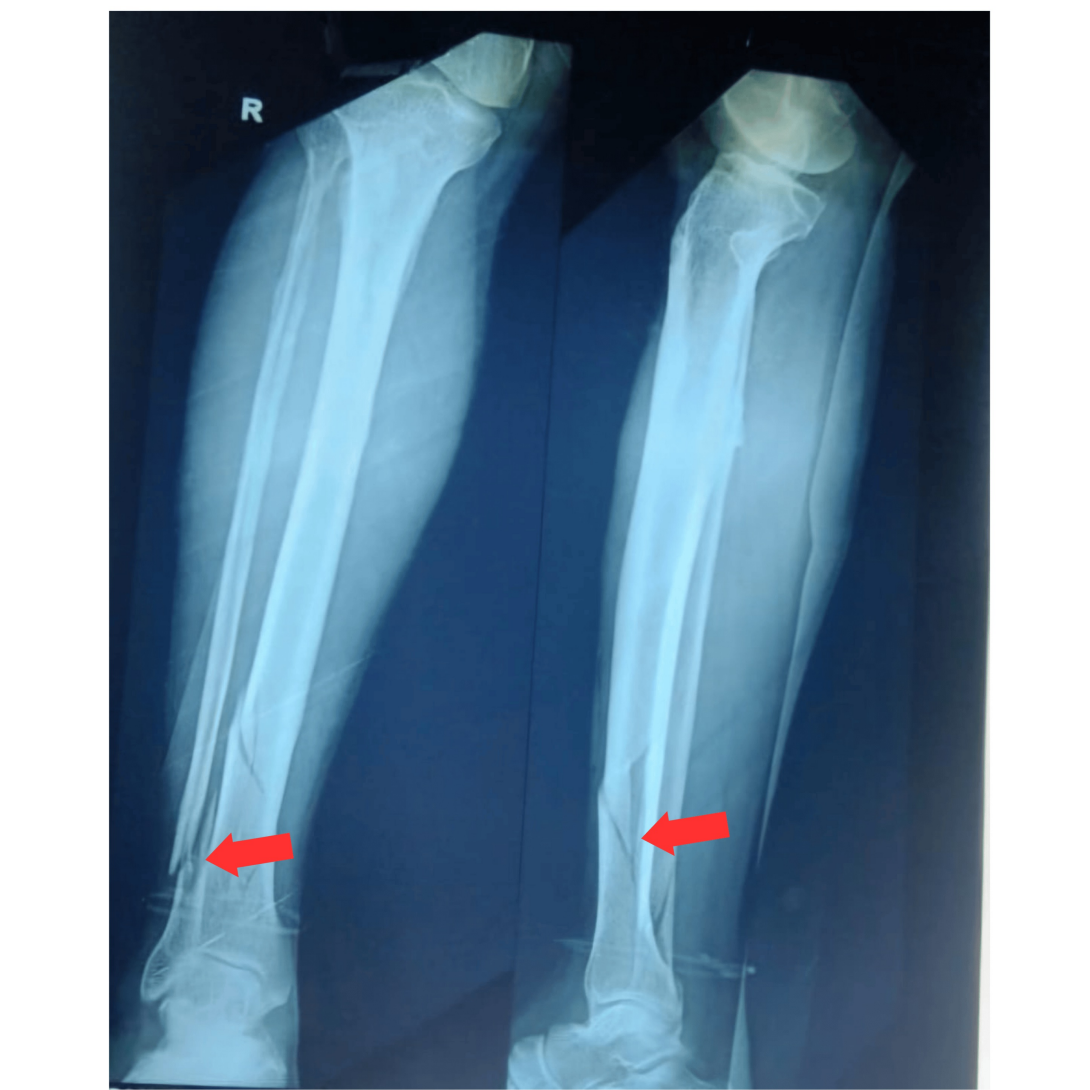
X-ray showing multiple leg fractures in a 37-year-old male trauma patient on admission to the emergency department. Arrows indicate multiple comminuted fractures of the right and left tibia and fibula.

Figure 1 presents the patient's leg X-ray, which shows multiple comminuted fractures of the right and left tibia and fibula. The patient’s comminuted fractures of the tibia and fibula increased the risk of fat embolism syndrome (FES), where fat droplets from the bone marrow enter the bloodstream and obstruct blood flow to the lungs, brain, and other vital organs. The patient’s compromised respiratory status increased this risk. FES typically develops within 24 to 72 hours post injury, manifesting as respiratory distress, hypoxemia, neurological changes, and a petechial rash. The combination of multiple bone fractures, chest trauma, and compromised respiratory function also increased the patient’s risk for developing acute respiratory distress syndrome (ARDS), characterized by widespread lung inflammation and worsening of his arterial hypoxemia. The pain associated with multiple fractures and rib injuries resulted in shallow breathing, worsening hypoventilation, and hypoxemia. The patient’s intoxicated state and respiratory instability delayed surgical intervention for the tibia and fibula fractures. There was concern that prolonged delay in fixation of the fractures would increase the risk of FES, infection, and/or non-union.

A trauma series of CT scans included the head, neck, chest, abdomen, and pelvis. The scans were negative for hemorrhage and fractures. Blood work showed that erythrocyte (RBC) count (2.71 x 10^6^/uL), hemoglobin (8.5 g/dL), and hematocrit (26.1%) were all low; mean corpuscular hemoglobin (MCH = 44 pg) and mean corpuscular volume (MCV = 109 fL) were high. The platelet count on admission was unmeasurable likely due to agglutination of the patient's platelets by the cold agglutinins. An unreliable measurement showed a platelet count of 10,000/uL, indicative of severe thrombocytopenia. A differential leukocyte (WBC) count was high and left-shifted, with numerous immature band neutrophils. Arterial blood gases were indicative of acute respiratory insufficiency. Blood coagulation factors were normal. We performed both direct and indirect antiglobulin (Coombs) tests, measured immunoglobulin concentrations in the serum, and C3 and C4 complement components. We discovered a positive direct and negative indirect antiglobulin test and a high IgM content. Cold agglutinins were found to be present and the patient's blood type was A+. Urinalysis showed oliguria, negative for protein and WBCs.

Table 1 presents the initial laboratory values for this patient on admission. The main concern was the patient's respiratory status; his low partial pressure of oxygen (PaO_2_) and high partial pressure of carbon dioxide (PaCO_2_) with low arterial pH were indicative of alveolar hypoventilation with acute uncompensated respiratory acidosis, in the absence of metabolic acidosis. The patient’s decreased chest wall movement, inadequate ventilation, and reduced ability to clear his respiratory tract secretions exacerbated the patient’s low respiratory rate and low oxygen saturation, increasing the risk of low oxygen delivery to vital organs and tissue hypoxia.

**Table 1:** Laboratory values of a 37-year-old male trauma patient on admission to the emergency department.

Test category	Value	Reference range
Coagulation tests		
Prothrombin time	13 seconds	11-13.5 seconds
Activated partial thromboplastin time	27 seconds	25-35 seconds
Urinalysis		
Specific gravity	1.018	1.005-1.030
pH	7.0 log M	6-7 log M
Leukocyte count	10 x 10^6^/uL	4-11 x 10^6^/uL
Neutrophils	84.0%	65-70%
Basophils	1.%	0.5-1%
Eosinophils	2%	1-4%
Lymphocytes	22%	20-40%
Monocytes	2%	2-8%
Blood alcohol level	65 mg/dL	30 mg/dL
Partial pressure of oxygen (PaO_2_)	60 mmHg	80-100 mmHg
Partial pressure of carbon dioxide (PaCO_2_)	54 mmHg	35-45 mmHg
Arterial pH	7.24	7.35-7.45

**Figure 2:**
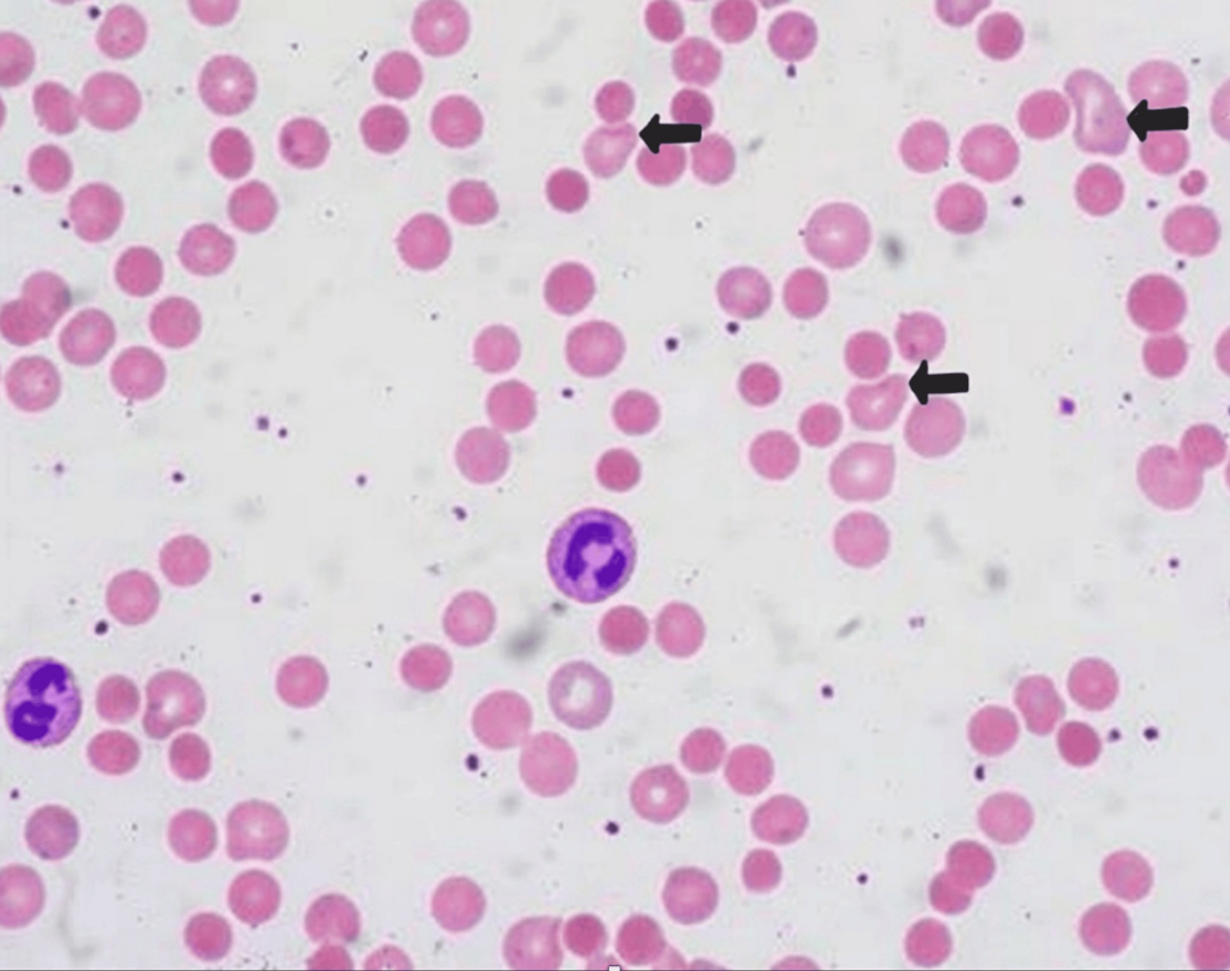
Blood smear showing agglutination in a 37-year-old male trauma patient. Arrows point to ghost cells representing hemolyzed erythrocytes, likely caused by cold agglutinins in the patient subjected to traumatic injuries associated with a motorcycle accident. The blood smear shows erythrocytes with pale intracellular staining at the locations indicated by the arrows. These ghost cells are likely a result of agglutination, which is thought to be produced by the presence of cold agglutinins. The pale intracellular staining is indicative of hemolysis, where the erythrocytes have been partially lysed, leaving behind empty or ghost cells (700x magnification).

Figure 2 shows the patient's blood smear on admission, demonstrating the presence of ghost cells indicative of hemolysis. In this trauma patient with the likelihood of acute blood loss due to multiple fractures, the presence of ghost cells and agglutination on a blood smear was thought to be highly significant. This clinical presentation suggested ongoing intravascular hemolysis, which could complicate the clinical outcome by worsening anemia, impairing circulation, and increasing the risk of tissue hypoxia and organ damage. This finding necessitated careful medical management to prevent further hemolysis, maintain adequate oxygen delivery, and address both the trauma and the underlying hematologic disorder.

Diagnosis

Because acute blood loss is usually associated with proportional loss of erythrocytes and plasma, severe anemia is not expected. The anemia found in this intoxicated patient immediately following a motor vehicle accident raised the suspicion of anemia associated with alcoholism. Anemia in alcoholics may arise from a combination of nutritional deficiencies, especially folate and iron, bone marrow suppression, direct alcohol-induced hemolysis, liver disease and/or cirrhosis, gastrointestinal bleeding, as well as direct effects of alcohol on red blood cell production and function. However, the direct Coombs test was positive, due to the presence of complement components such as C3d on the patient’s RBCs, thereby suggesting the diagnosis of cold agglutinin disease. This diagnosis, coupled with the acute blood loss from the motor vehicle accident, likely explains the observed abnormalities in the patient's RBC parameters, including low hemoglobin and hematocrit levels, as well as elevated MCH and MCV values. The combination of these factors may have contributed to impaired oxygen delivery to tissues, manifesting as difficulty breathing.

Treatment

The patient was treated with intravenous immunoglobulin (IVIG) at 1 mg/kg to decrease cold agglutinins. Blood transfusion was not administered because the patient's condition was not immediately life-threatening and there was concern about untoward effects associated with combining intravenous blood products with IVIG. Additionally, there was concern about a blood transfusion inducing internal hemorrhages because of thrombocytopenia. In this polytrauma patient with cold agglutinin disease and a critically low platelet count, below 10,000/µL or unmeasurable, there was concern about a blood transfusion inducing internal hemorrhaging. A platelet count below 10,000/µL represents severe thrombocytopenia, which significantly increases the risk of spontaneous bleeding, including internal hemorrhage. In the presence of a high titer of cold agglutinins, agglutination is not limited to RBCs; it can also affect platelets. Platelet agglutination makes them dysfunctional, further reducing the effective platelet count. This exacerbated the risk of hemorrhage in this patient because of trauma with the potential for ongoing bleeding and the need for surgery on multiple bone fractures. It is noteworthy that blood transfusions in such patients must be handled cautiously because the introduction of cold-stored blood products can exacerbate hemolysis if the transfused blood cools the patient or if cold agglutinins react with the transfused RBCs. Another concern was that transfusing platelets or other blood components that would have been stored at cold temperatures could trigger further agglutination of the patient’s platelets, leading to an acute drop in functional platelet levels, further increasing the risk of bleeding. Table 2 summarizes the key hematology laboratory findings before and after treatment, demonstrating the effectiveness of the IVIG treatment in reversing the patient's anemia.

**Table 2:** Effects of intravenous immunoglobulin (IVIG) treatment on hematological laboratory values in a 37-year-old trauma patient with high levels of cold agglutinins. MCV = mean corpuscular volume; MCH = mean corpuscular hemoglobin; RBC = red blood cell (erythrocyte).

Test category	Before treatment	After treatment	Reference range
Hematocrit	26.10%	34.20%	38.8-50.0%
Hemoglobin	8.5 g/dL	11.5 g/dL	13.5-17.5 g/dL
MCV	109 fL	91 fL	80-100 fL
MCH	44 pg	30 pg	27-32 pg
RBC count	2.71 x 10^6^/uL	4.80 x 10^6^/uL	4.5-5.5 x 10^6^/uL

## Discussion

In a trauma patient under the influence of alcohol after a motor vehicle accident with multiple fractures, we strongly suspected cold agglutinins as the cause of the deranged RBC parameters due to the combination of acute blood loss and the cold environment in which the accident occurred. Given these factors, IVIG treatment was initiated at 1 mg/kg, not only as the standard of care but also as a strategic diagnostic tool. IVIG, which contains antibodies capable of neutralizing harmful autoantibodies, was expected to reverse the RBC derangements specifically if cold agglutinin disease (CAD) was present. The rationale was that a significant improvement in RBC parameters following IVIG administration would confirm the diagnosis. Indeed, after treatment, the patient's hemoglobin (Hb), hematocrit (Hct), and RBC count increased, and the MCV and MCH normalized, leading to marked clinical improvement. The patient’s respiratory function improved, anemia symptoms were alleviated, and the patient subsequently underwent management for the polytrauma injuries. This case underscores the importance of considering CAD in trauma patients with unexplained anemia and respiratory distress, particularly when there is potential exposure to cold. Early intervention with IVIG can both confirm the diagnosis and significantly enhance patient outcomes.

Cold agglutinins are autoantibodies, predominantly of the IgM class, which attach to RBC surface antigens and trigger hemolysis [6]. Under physiological conditions, cold agglutinins aid in immune defense against pathogens, especially in cold environments. When the production of cold agglutinins increases or their specificity changes, they can cause anemia and symptoms of fatigue, pallor, and dyspnea. Agglutination can restrict blood flow, thereby exacerbating tissue ischemia and impacting organ function. In trauma scenarios, cold agglutinins can complicate the interpretation of lab results and clinical assessments [7]. This may result in inaccuracies in blood tests, e.g., RBC counts and Hb levels. If agglutination occurs during specimen handling or transport under cold conditions, the cold agglutinins cause RBCs to clump, artificially elevating RBC counts and Hb values. Such effects may obscure the actual severity of anemia or hemolysis, potentially leading to diagnostic mistakes and inappropriate medical interventions [8,9].

IVIG is a blood product that contains antibodies, which can help to neutralize the harmful effects of the patient's antibodies. Because IgM, in terms of size and mass, are the largest human antibodies, their hexameric (or pentameric) forms contain antigen-binding sites sufficiently wide apart to overcome the distance imposed by RBCs in suspensions such as plasma, thereby permitting spontaneous agglutination [10,11]. Small concentrations of cold agglutinins are prevalent in healthy humans, and temperatures above 4°C render them inactive. Pathological cold agglutinins often react between 28°C and 31°C. Cryoglobulins may also influence the number of RBCs found at low temperatures. Cryoglobulins are circulating immunoglobulins that become insoluble and precipitate at temperatures between 4°C and 37°C, interfering with the accuracy of automated blood cell counters and primarily causing pseudoleukocytosis and pseudothrombocytosis [10,11]. Cryoglobulin particles that pass through the analyzer’s aperture may be counted as WBCs or platelets if they match their size, structure, and form. Cryoglobulins, unlike cold agglutinins, often have a minor impact on RBC count and Hb measurement. Consequently, cold agglutinins and cryoglobulins interfere differently with blood cell counts, but the laboratory process for analyzing such samples is identical in both instances.

The present case involves a 37-year-old man with erroneously low RBC counts and a disparity between Hb and Hct values. This prompted a search for evidence of increased expression of cold agglutinins. Clinical laboratory analyzers directly measure RBC, Hct, and Hb while MCV, MCH, and mean corpuscular hemoglobin concentration (MCHC) are calculated based on the measured values [12]. The present patient’s blood produced RBC microaggregates, which led to the low RBC count. The analyzer counts the microaggregates as WBCs or single RBCs, whereas massive aggregates are excluded. These factors result in an erroneously low RBC count and, as a result, aberrant values for the other measured and calculated parameters [3]. Using three measured values, the analyzer calculates MCH and MCHC indices. MCH is calculated as the quotient of Hct and RBC count (MCH, pg = Hct/RBC), whereas MCHC is calculated as the quotient of Hb and Hct (MCHC, g/L = Hb/Hct). The computation of MCHC serves as a quality control mechanism. Elevated MCHC indicates sample or analytical mistakes (hemolysis, lipemia, agglutination, etc.). Modern automated hematology analyzers signal potential errors with flags [13]. In the present case, the MCHC level was highly elevated, and the analyzer flagged RBC agglutination. Typically, agglutination can be detected visually, but a blood smear study best confirms it, as shown in Figure 2.

After warming one of the patient's blood specimens to 37°C, we observed relative normalization of the RBC parameters, as recommended [14]. Warming the sample causes the IgM antibody to elute from the surface of cells, allowing agglutinated RBCs to separate. Nikousefat et al. report a female patient with a low RBC count, incompatible Hct and Hb, and increasing RBC indices assessed on an automated Sysmex analyzer (Hyogo, Japan). The patient was diagnosed with coronary artery disease, and warming the sample to 37°C led to accurate results [15]. Using the ABX Pentra 80 hematology analyzer (HORIBA Medical, Kyoto, Japan), Ercan et al. reported comparable results when they measured the cold agglutinin antibody titer before and after proper sample transit in a heated container with immediate analysis [16]. Kakkar used an Advia 60 hematology analyzer (Siemens Healthineers, Forchheim, Germany) [17]. WBC counts and Hb were reported to be unaltered by cold agglutinins when Hb was directly tested by lysing RBCs [10]. Artificial inflation of the Hb concentration might account for this in hemolyzed blood samples. Because platelets can undergo auto-agglutination, platelet counts and related platelet indices can also be mismeasured in the presence of cold agglutinins. Yasar et al. report a scenario wherein platelet count and mean platelet volume were unmeasurable, but were quantified normally after warming the sample to 37°C [18].

Cold agglutinins associated with trauma-related anemia are often found in the absence of a diagnosis of overt CAD. CAD is typically manifested as transient hemolysis triggered by cold exposure during the traumatic event rather than chronic autoimmune hemolysis. Nevertheless, the diagnosis of CAD must be considered whenever there is (i) hemolytic anemia with symptoms such as fatigue, pallor, jaundice, and dark urine; (ii) acrocyanosis of extremities (fingers, toes, ears, nose) when exposed to cold temperatures; or (iii) Raynaud's phenomenon, where fingers and toes turn white, blue, and then red upon exposure to cold or stress. Laboratory testing to confirm the diagnosis of CAD involves, firstly, a complete blood count (CBC) to detect anemia. Elevated reticulocytes indicate increased red blood cell production as a response to hemolysis. A peripheral blood smear will demonstrate agglutinated RBCs. The direct antiglobulin test (DAT or Coombs test) is typically positive for complement C3d but not IgG in CAD. Cold agglutinin titer must be performed to directly measure the level of cold agglutinins; titers > 1:64 are usually significant [19]. Serum haptoglobin, with low levels indicating hemolysis; lactate dehydrogenase, with high levels indicating hemolysis; and bilirubin levels, with elevated indirect bilirubin indicating increased breakdown of RBCs are all confirmatory of CAD.

In the present case, a positive Coombs test showed the presence of IgG and C3 in erythrocytes. In a study of CAD in 58 patients, anti-C3d antibody positivity was found in 74%, anti-C3d + anti-IgG positivity in 22%, and anti-IgG positivity in 3.4%. Aside from standard laboratory analysis issues, the presence of cold agglutinins makes blood type detection challenging. Lodi et al. described a case of a 48-year-old patient whose blood group could not be established likely because of analytical difficulties associated with the presence of cold agglutinins [20]. The patient died after receiving an emergency transfusion of universal RBCs. The presence of cold agglutinins in the patient's blood made it impossible to determine their blood type accurately, necessitating the use of universal donor RBCs. The patient died after receiving the emergency transfusion likely because of a severe hemolytic reaction caused by the cold agglutinins reacting with the transfused RBCs, leading to acute hemolysis and subsequent organ failure and other complications.

## Conclusions

When a trauma patient presents with low Hb and Hct, the suspicion of cold agglutinin disease should be raised only when this is accompanied by a disparity, specifically when the low Hb and Hct are coupled with elevated MCH and MCV. This combination, rather than low Hb and Hct alone, warrants consideration of cold agglutinins as a potential cause of the anemia. In cases involving multiple traumatic injuries, the initial presentation of Hb and Hct levels may appear relatively normal due to the proportionate loss of both red blood cells and plasma during acute blood loss. However, the presence of cold agglutinins can lead to abnormal agglutination and sequestration of RBCs. Agglutination can cause falsely low Hb and Hct measurements, which can obscure the true blood volume status. Therefore, detecting cold agglutinins is crucial for accurately diagnosing and managing anemia in patients with traumatic polyinjuries.
